# APRIL Drives a Coordinated but Diverse Response as a Foundation for Plasma Cell Longevity

**DOI:** 10.4049/jimmunol.2100623

**Published:** 2022-09-01

**Authors:** Sophie Stephenson, Matthew A. Care, Gina M. Doody, Reuben M. Tooze

**Affiliations:** * Division of Haematology and Immunology, Leeds Institute of Medical Research, University of Leeds, Leeds, United Kingdom; and; † Bioinformatics Group, School of Molecular and Cellular Biology, University of Leeds, Leeds, United Kingdom

## Abstract

Ab-secreting cells survive in niche microenvironments, but cellular responses driven by particular niche signals are incompletely defined. The TNF superfamily member a proliferation-inducing ligand (APRIL) can support the maturation of transitory plasmablasts into long-lived plasma cells. In this study, we explore the biological programs established by APRIL in human plasmablasts. Under conditions allowing the maturation of ex vivo– or in vitro–generated plasmablasts, we find that APRIL drives activation of ERK, p38, and JNK, accompanied by a classical NF-κB response and activation of the AKT/FOXO1 pathway. Time-course gene expression data resolve coordinated transcriptional responses propagated via immediate early genes and NF-κB targets and converging onto modules of genes enriched for MYC targets and metabolism/cell growth–related pathways. This response is shared between APRIL and an alternate TNF superfamily member CD40L but is not a feature of alternative niche signals delivered by IFN-α or SDF1. However, APRIL and CD40L responses also diverge. CD40L drives expression of genes related to the activated B cell state whereas APRIL does not. Thus, APRIL establishes a broad foundation for plasma cell longevity with features of cellular refueling while being uncoupled from support of the B cell state.

## Key Points

- Survival signals drive distinct gene expression programs in human plasmablasts.
- APRIL and CD40L share activation of cell growth–related gene programs.
- APRIL-driven growth programs are uncoupled from support of the B cell state.

## Visual Abstract

**Figure:**
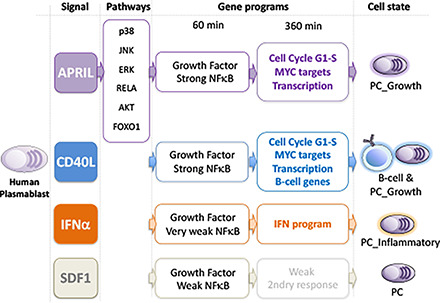

## Introduction

The survival of plasma cells (PCs) is dependent on specific niche conditions. On the one hand, this allows the maintenance of long-lived humoral immunity, and on the other hand it provides a flexible mechanism for limiting the PC pool (1). Additionally, both the nature of the differentiating B cell, the type of signal driving differentiation, and the nature of the niche in which the Ab-secreting cell (ASC) eventually survives as a long-lived PC (also referred to as memory PCs) may convey functional specialization (2, 3). Several niche factors have been defined that may contribute to the survival of an ASC and allow the maturation from the transitional plasmablast (PB) state, which couples proliferation and acquisition of secretory capacity to the quiescent but long-lived PC state (1, 4–6). However, relatively little is known regarding the specific signaling pathways and downstream transcriptional responses to individual niche signals in ASCs. In this study, we use a model system that allows the in vitro generation of long-lived human PCs to study the response of human ASCs to the niche factor APRIL, as the cells initiate the final differentiation step to the quiescent PC state.

APRIL belongs to the TNF superfamily (TNFSF). This superfamily, along with its cognate receptors, includes several critical regulators of B cell survival, activation, and commitment to the ASC differentiation fate (7). APRIL and its most closely related TNFSF member BAFF share partially overlapping receptors in BCMA (TNFRSF17), TACI (TNFRSF13B), and BAFFR (TNFRSF13C) (8). These receptors are themselves regulated during differentiation of B cells, such that BAFFR dominates in resting B cells, allowing effective signaling from BAFF but not APRIL, whereas BCMA predominates in PBs and PCs. TACI bridges these patterns with expression peaking during activation (7). This provides the potential for preferential responses from APRIL rather than BAFF at later stages of differentiation. Further layers of regulation operate both in relationship to the shedding of surface receptors and the extent of oligomerization of the ligands (9). Notably BCMA, the primary receptor for APRIL, can be cleaved and shed from the cell surface by the action of γ-secretase, which has been identified as a limiting factor for APRIL responses in PC populations in vivo and in cell lines in vitro (10).

The importance of APRIL/BCMA signals to PC survival has been demonstrated in murine models (11, 12), as well as in humans where targeting has been explored as a therapeutic avenue in rheumatological conditions (13, 14). Functionally, in murine PCs BCMA signals are thought to support survival through induction of MCL1 (15). BCMA signals can also support myeloma cell survival in vitro and in vivo (16). Studies in B cells have demonstrated the activation of MAPK pathways and classical NF-κB responses following stimulation with BAFF, and similar responses are observed to APRIL in PC myeloma cell lines (7, 17). In heterologous expression systems BCMA signaling has been shown to have the potential to activate p38 and JNK MAPK pathways alongside classical NF-κB responses (18). Indeed, in PC neoplasia, mutations affecting the NF-κB pathway are frequent and associated with progression and independence from niche survival signals (19–21).

In this study, we have addressed the question of how primary human ASCs respond to the APRIL niche signal focusing on the responses that occur at the transition between PBs and quiescent PCs. Under conditions that efficiently promote the survival of both in vitro–generated and ex vivo–derived human PBs, APRIL delivers a complex signal inducing gene expression features related to cell growth programs of B cell activation to establish a broad foundation for PC survival. The response differs significantly from other niche signals such as IFN-α that promote PC survival independent of such growth-related programs. In contrast, APRIL and CD40L responses share many similarities in gene regulation, but APRIL differs from CD40L in being uncoupled from support of the B cell state. Thus, APRIL provides a selective signal for PC survival in differentiating PBs.

## Materials and Methods

### Reagents

Reagents used included the following: IL-2 (Miltenyi Biotec); IL-21, IL-6, and SDF1 (PeproTech); IFN-α (Sigma-Aldrich); multimeric APRIL H98 and multimeric CD40L (AdipoGen Life Sciences); goat anti-human IgM and IgG F(ab′)_2_ fragments (Jackson ImmunoResearch); lipid mixture 1, chemically defined (200×) and MEM amino acids solution (50×) (Sigma-Aldrich); and L-685,458 (γ-secretase inhibitor [GSI]) (Tocris).

### Donors and cell isolation

Peripheral blood was obtained from healthy donors after informed consent. The number of donors per experiment is indicated in the figure legends, with each symbol representing a different donor. Mononuclear cells were isolated by Lymphoprep (Abbott) density gradient centrifugation. Total B cells were isolated by negative selection with a memory B cell isolation kit (Miltenyi Biotec).

Peripheral blood samples from anonymous donors were obtained on day 6–8 after influenza vaccination (2017–2018). BCMA-positive cells were isolated using a combination of BCMA-biotinylated Ab and anti-biotin microbeads (Miltenyi Biotec).

### Cell cultures

Cells were maintained in IMDM supplemented with GlutaMAX and 10% heat-inactivated FBS (Invitrogen); lipid mixture 1, chemically defined and MEM amino acids solution (both at 1× final concentration) were added from day 3 onwards.

For day 0 to day 3, B cells were cultured in 24-well plates at 2.5 × 10^5^/ml with IL-2 (20 U/ml), IL-21 (50 ng/ml), and F(ab′)_2_ goat anti-human IgM and IgG (2 µg/ml) on gamma-irradiated CD40L-expressing L cells (6.25 × 10^4^/well).

For day 3 to day 6, at day 3, cells were detached from the CD40L L cell layer and reseeded at 1 × 10^5^/ml in media supplemented with IL-2 (20 U/ml) and IL-21 (50 ng/ml).

For day 6 to day 13, at day 6, cells were harvested and seeded at 1 × 10^6^/ml in media supplemented with IL-6 (10 ng/ml), IL-21 (10 ng/ml), GSI (100 nM), and multimeric APRIL (100 ng/ml unless otherwise stated) or soluble CD40L (sCD40L; 100 ng/ml).

For culture of ex vivo cells, following isolation cells were cultured in media containing IL-6 (10 ng/ml) and IL-21 (10 ng/ml) for 24 h. Cells were then harvested and transferred into media containing IL-6 (10 ng/ml) and either multimeric APRIL (100 ng/ml) and GSI (100 nM), or IFN-α (100 U/ml) for 14 d. Half of the media was replenished after 7 d.

### Flow cytometric analysis

Cells were analyzed using four- to six-color direct immunofluorescence staining on a CytoFLEX LX or S (Beckman Coulter) flow cytometer. Abs used included CD19 PE (LT19) and CD138 allophycocyanin (44F9) (Miltenyi Biotec); CD20 e450 (2H7) (eBioscience); and CD27 FITC (M-T271) and CD38 PE-Cy7 (HB7) (BD Biosciences). Controls were isotype-matched Abs or fluorescence-minus-one controls. Dead cells were excluded by 7-aminoactinomycin D (BD Biosciences). Absolute cell counts were performed with CountBright beads (Invitrogen). Cell populations were gated on forward scatter and side scatter profiles for viable cells determined independently in preliminary and parallel experiments. Analysis was performed with FlowJo version 10 (BD Biosciences) and Prism 8/9 (GraphPad Software). Statistical analysis performed was either a two-tailed paired *t* test or repeated measures one-way ANOVA, Tukey’s multiple comparisons test.

### Protein analysis

At the indicated time points, cells were lysed in Laemmli buffer. For cytoplasmic/nuclear protein samples, proteins were extracted using a cytoplasmic and nuclear extraction kit (Boster Bio), and protein concentration was determined by bicinchoninic acid assay (Boster Bio). Samples were separated by SDS-PAGE and transferred to nitrocellulose membranes. Proteins were detected by ECL (SuperSignal West Pico PLUS, Thermo Fisher Scientific) and visualized on a ChemiDoc (Bio-Rad) or film. Protein bands were quantitated using Image Lab 6.0.1 software (Bio-Rad) or ImageJ.

Abs used were p-AKT, AKT, p-ERK1/2, ERK1/2, p-FOXO1/3/4, FOXO1, FOXO3, p-JNK, JNK, p-p38, p38, MYC, RELA, p-SQSTM1 T269/S272, and SQSTM1 (CST); tubulin (Merck); H3 (Abcam), BLIMP-1 (R23) (22); and goat anti-mouse HRP and goat anti-rabbit HRP (Jackson ImmunoResearch).

### ELISPOT

Influenza-specific Ig was detected as previously described (23). Inactivated influenza vaccine manufactured by Sanofi Pasteur MSD was used for coating plates.

For detection of human IgG secretion, a human ELISpot^BASIC^ IgG kit (Mabtech) was used. The assay was performed as described in the manufacturer’s protocol, and 1000/2000 cells were added as indicated in the figures. Cells were incubated on plates for 16–20 h in IMDM containing either standard amounts of IL-6 and IL-21 (ex vivo), or IL-6 with either IFN-α or APRIL/GSI (in vitro + 14 d).

### Gene expression data acquisition and analysis

Gene expression datasets were generated from differentiating PBs (day 7). At day 6, PBs from *n* = 4 (APRIL time course) or *n* = 3 (niche time course) healthy donors were seeded at 1 × 10^6^/ml in phenol red–free IMDM supplemented with 0.5% heat-activated FBS, IL-6 (10 ng/ml), IL-21 (10 ng/ml), and GSI (100 nM) and incubated for 20 h. Multimeric APRIL (100 ng/ml) was then added. For the APRIL time course data a pretreatment sample (0 min) and posttreatment samples were removed at +30, +60, +120, and +360 min. For comparison between niche signals a pretreatment sample (0 min) and posttreatment samples were removed at +60, and +360 min following treatments with multimeric APRIL (100 ng/ml), sCD40L (100 ng/ml), SDF1(10 ng/ml), or IFN-α (100 U/ml).

RNA was obtained using TRIzol (Invitrogen) and sequencing libraries were generated with a TruSeq stranded total RNA human/mouse/rat kit (Illumina). Libraries were sequenced on the NextSeq500/NovaSeq6000 platforms (Illumina), using either 76-bp single-end sequencing (NextSeq500; APRIL time course) or 150-bp paired-end sequencing (NovaSeq6000; niche time course), with fastq files subject to initial quality assessment, trimming, alignment, and annotation. Transcript abundance was estimated using RSEM v1.3.0 and processed using DESeq2 to determine differential gene expression. Expression datasets are available with Gene Expression Omnibus accession number GSE205101 (https://www.ncbi.nlm.nih.gov/geo/query/acc.cgi).

### Network analysis

For the bulk APRIL time course network, transcripts differentially expressed across the time series data (DESeq2 likelihood ratio test false discovery rate [FDR] < 0.01) were retained. For the niche signal time course network, transcripts that were either differentially expressed across the time series (DESeq2 likelihood ratio test FDR < 0.05) or differentially expressed between any pair of conditions at any time point (DESeq2; FDR < 0.05) were retained. Both datasets were merged per gene by taking the median value for transcript sets with a Pearson correlation ≥0.2 and the maximum value for those with a correlation <0.2. This resulted in a 4615 × 20 matrix (APRIL time course) and a 6574 × 27 matrix (niche time course). Parsimonious Gene Correlation Network Analysis (PGCNA2) (-n 1000, -b 100) was applied (24), giving networks with 16 modules (APRIL time course) and 15 modules (niche time course), respectively. The median expression per time point was visualized as *Z* scores mapped onto the network. The top 10 genes per module by network strength were used to generate module expression values using median *Z* scores and visualized as a hierarchically clustered heatmap.

### Network, gene lists, and signature enrichment availability

Interactive networks and extended data are available at https://mcare.link/STC-APRIL.

### Gene signature data and enrichment analysis

A set of 40,686 signatures was generated by merging Gene Ontology and gene signatures as previously described (24). Enrichment of gene lists for signatures was assessed using a hypergeometric test, in which the draw is the gene list genes, the successes are the signature genes, and the population is the genes present on the platform.

### Heatmap visualizations

The gene expression data and gene set enrichment results were both visualized using the Broad GENE-E package (https://software.broadinstitute.org/GENE-E/). For visualization of expression data, the module expression values were visualized on a relative *Z* score scale. For gene set enrichment the signatures were filtered (FDR of <0.1 and ≥5 and ≤1500 genes for the signature sets, selecting the top 15 most significant signatures per module) and the enrichment/depletion *Z* scores were visualized. In both cases the data were hierarchically clustered (Pearson correlations and average linkage).

### Ethical approval

Approval for this study was provided by UK National Research Ethics Service via the Leeds East Research Ethics Committee (approval reference no. 07/Q1206/47).

## Results

### APRIL supports in vitro PC survival that is enhanced by γ-secretase inhibition

We have previously defined conditions that allowed the generation of long-lived PCs in vitro both using stromal support and independent of stroma using type 1 IFN or TGF-β–mediated survival signals (5, 6). These conditions allowed PC survival in the absence of defined TNFSF signaling, and in the absence of detectable NF-κB–mediated transcriptional response as assessed by gene signature analysis (3, 5). This was notable because PC malignancies frequently show NF-κB pathway deregulation and the TNFSF member APRIL is thought to provide a key PC niche signal acting at least in part through the NF-κB pathway (7, 25, 26). We therefore aimed to take advantage of our model system to analyze the impact of APRIL signaling in more detail as the PC completed differentiation.

**FIGURE 1.**
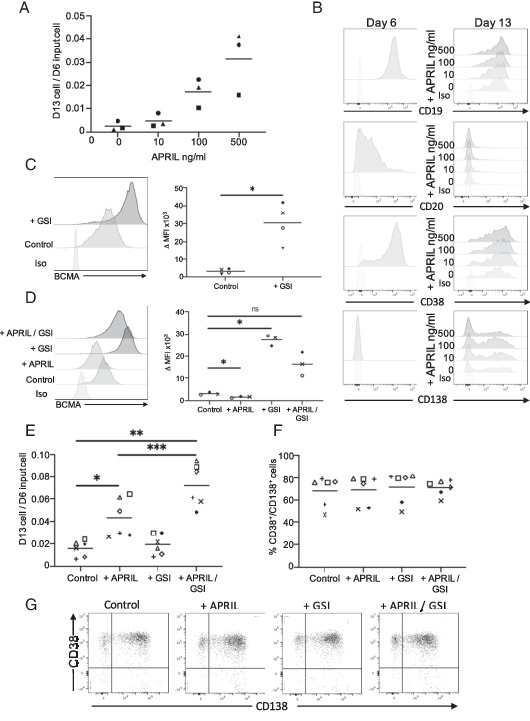
APRIL and γ-secretase inhibition support in vitro PC differentiation and survival. (**A**) APRIL dose response showing recovered cell number at day 13 of in vitro culture; *y*-axis shows fraction of cells recovered at day 13 (D13) per input cell at day 6 (D6), and *x*-axis shows concentration of multimeric APRIL (ng/ml) from day 6 (all conditions included a standard dose of IL-6 and IL-21); data are shown for three donors (symbols). (**B**) Representative flow cytometry plots for selected Ags. Left panel, Phenotype at day 6, before the addition of APRIL. Right panel, phenotype at day 13 following culture in IL-6, IL-21, and APRIL. Results are shown for each APRIL concentration equivalent to (A) and Ags highlighted below each panel with isotype (Iso) shown on the bottom plot. (**C**) Impact of γ-secretase inhibitor (GSI) on BCMA expression. Left panel, Representative flow cytometry data for surface BCMA expression at day 7 following treatment with indicated conditions. The bottom plot represents isotype (Iso) staining. Right panel, ΔMFI (×10^3^) for BCMA expression against isotype control. Data are shown for four donors (paired *t* test: *p < 0.05). MFI, mean fluorescence intensity. (**D**) Impact of APRIL treatment on BCMA expression in the presence or absence of GSI. Left panel, Representative flow cytometry data for surface BCMA expression at day 13 following treatment with indicated conditions. Right panel, ΔMFI (×10^3^) for BCMA expression against isotype control. Data shown are for three donors (repeated measures one-way ANOVA test: *p < 0.05). (**E**) Cell number recovered after APRIL stimulation from day 6 to day 13 of in vitro culture under conditions indicated (*x*-axis); *y*-axis shows fraction of cells recovered at day 13 (D13) per input cell at day 6 (D6) (repeated measures one-way ANOVA test: *p < 0.05, **p < 0.01, ***p < 0.001). (**F**) Percentage of CD38^+^/CD138^+^ cells observed at day 13 in samples cultured as in (E). (**G**) Representative scatterplots of CD38 versus CD138 expression of a single donor at day 13, with culture conditions indicated above each panel. Data shown in (E) and (F) are representative of six donors. In (C)–(E), control conditions are media plus a standard dose of IL-6 and IL-21. Individual donors are indicated by unique symbols that are consistent across all figures.

We first used the ability of APRIL to support PC survival as a functional indicator of effective signaling. Initially performing a dose response, we observed that PC survival could be effectively supported by APRIL in multimeric form but required significant quantities (Fig. 1A). Under these conditions the phenotype of the differentiated cells was consistent with an early PC state showing strong CD38 expression, partial upregulation of CD138, and loss of CD20 (Fig. 1B). Recently it was observed that BCMA, the primary surface receptor for APRIL, expressed on ASCs was subject to active proteolytic shedding and that this was dependent on γ-secretase activity (10). We therefore tested whether this effect was observed in ASCs in the model system. Indeed, γ-secretase inhibition substantially augmented the expression of cell surface BCMA during in vitro differentiation. A 6-fold enhancement of BCMA expression was observed following γ-secretase inhibition (Fig. 1C), and this increase was largely maintained in the presence of APRIL stimulation (Fig. 1D).

The enhancement of surface BCMA expression following γ-secretase inhibition translated into a significant increase in the impact of APRIL on in vitro PC survival (Fig. 1E). This increase in viability was associated with a generally similar phenotype of cell populations (Fig. 1F, 1G). Thus, the APRIL-mediated survival benefit for PC populations in vitro can be enhanced by inhibition of BCMA shedding in a fashion consistent with the model proposed by Laurent et al. (10) and providing further evidence that surface shedding is an intrinsic feature limiting BCMA signals at the PB to PC transition.

### APRIL support for ex vivo PB survival is enhanced by γ-secretase inhibition

**FIGURE 2.**
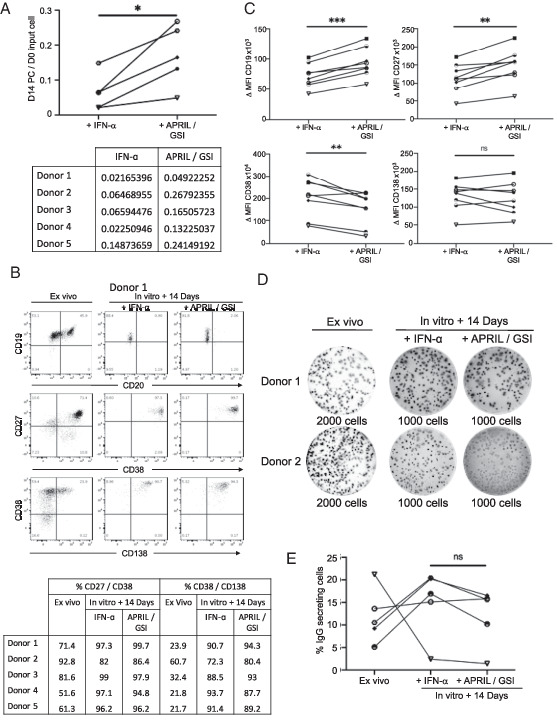
APRIL supports ex vivo PB maturation. (**A**) Comparison of cell recovery after 14 d of in vitro culture for ex vivo BCMA^+^ PBs isolated at day 7 after influenza vaccination cultured in IL-6 and either IFN-α or APRIL/GSI conditions (*x*-axis); *y*-axis shows PCs at day 14 (D14) per input cell at day 0 (D0) (paired *t* test: *p < 0.05). Data shown are for five donors (symbols), with tabulated results shown below. (**B**) Representative phenotypes of cells isolated ex vivo at day 7 after influenza vaccination left panels, or after 14 d of in vitro culture (equivalent to day 21 postvaccination) in IL-6 with IFN-α (middle) or APRIL/GSI (right). Scatterplots from top to bottom show CD19/CD20, CD27/CD38, and CD38/CD138 as indicated, with tabulated results shown below. (**C**) Differential expression for Ags assessed in (B), shown in order CD19 (upper left), CD27 (upper right), CD38 (lower left), and CD138 (lower right) as ΔMFI against isotype control (*y*-axis, ×10^3^ or ×10^4^ as indicated) for IFN-α (left) or APRIL/GSI (right) of each panel (*x*-axis). Each donor is identified with a unique symbol (paired *t* test: **p < 0.01, ***p < 0.001; ns, not significant). Data shown are for eight donors. MFI, mean fluorescence intensity. (**D** and **E**) Equivalent Ig secretion is supported by either IFN-α or APRIL/GSI conditions. Representative ELISPOT results are shown for two independent donors from ex vivo–isolated cells at day 7 after influenza vaccination (left) or after 14 d of in vitro culture with IL-6 and either IFN-α (middle) or APRIL/GSI (right), equivalent to day 21 after influenza vaccination (D) and quantitation (E) shown for five donors.

Because the combination of APRIL and γ-secretase inhibition provided an effective condition for in vitro–derived PB/PC transition, we next sought to determine whether this would also provide support for ex vivo PBs. We therefore isolated PBs from five donors following seasonal influenza vaccination at day 7 of the vaccine response and transferred these cells into survival conditions with either IFN-α or APRIL and γ-secretase inhibition. Two weeks later we assessed the phenotype, number, and secretory function of the PC population (Fig. 2). We compared samples at day 14 after in vitro culture to the baseline ex vivo cell state immediately after purification. Between the two treatment groups, the number of viable cells at 2 wk was significantly greater in the presence of APRIL and γ-secretase inhibition than in the presence of IFN-α (Fig. 2A). Phenotypically the conditions were similar in generating CD19^lo^CD27^hi^ CD38^hi^ and CD138^hi^ PCs (Fig. 2B, Supplemental Fig. 1A). However, consistently in the presence of APRIL and γ-secretase inhibition, the expression level of CD19 and CD27 was higher and the CD38 expression lower (Fig. 2C). The cell populations generated were indistinguishable at the level of per cell secretion of IgG as assessed by ELISPOT and included influenza vaccine–specific ASCs (Fig. 2D, 2E, Supplemental Fig. 1B, 1C). We noted that one of five donors who showed the highest percentage of IgG-secreting cells initially after purification subsequently generated the lowest percentage of IgG-secreting cells after 14 d of in vitro culture with an equivalent decline in both culture conditions. This response correlated with a higher percentage of CD38^+^/CD138^+^ cells immediately after extraction, but the reason for the differential survival of IgG-secreting cells in this context has not been fully determined. We conclude that IFN-α and APRIL can each promote survival and maturation of ex vivo PBs sustaining a similar population of PCs in terms of Ab secretion and phenotype. The subtle but reproducible differences in surface phenotype observed for in vitro–differentiated PCs under distinct niche conditions supports the contention that intracellular signals activated by the survival niche in which a PB matures impact the functional state of the resulting PCs.

### APRIL drives MAPK, NF-κB, and AKT/FOXO pathway activation

**FIGURE 3.**
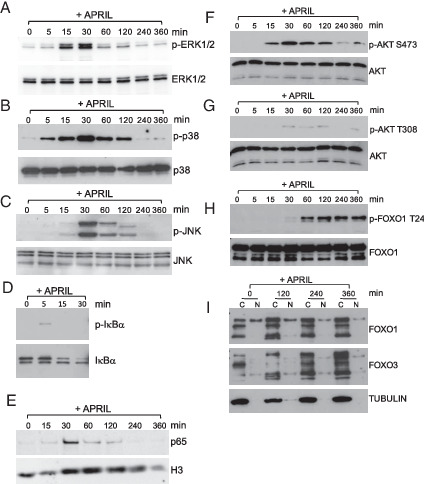
PB signaling responses to APRIL. (**A**) Time course of ERK1/2 phosphorylation induced after stimulation of day 7 PBs with APRIL. Upper panel shows detection of p-ERK1/2 (p-ERK) after stimulation with APRIL for the indicated time points of *t* = 0 (unstimulated), 5, 15, 30, 60, 120, 240, and 360 min. Total ERK1/2 loading control is shown below. (**B**) Time course of p38 phosphorylation induced by APRIL. Upper panel shows detection of p-p38 after stimulation with APRIL for the time course as in (A). Total p38 loading control is shown below. (**C**) Time course of JNK phosphorylation induced by APRIL. Upper panel shows detection of p-JNK1/2 (p-JNK) after stimulation with APRIL for the time course as in (A). Total JNK loading control is shown below. Western blots shown in (A)–(C) are representative of six donors. (**D**) Time course of IκBα phosphorylation after stimulation of day 7 PBs with APRIL. Upper panel shows p-IκBα and lower panel shows total IκBα after stimulation with APRIL for the indicated time points of *t* = 0 (unstimulated) 5, 15, and 30 min. Data are representative of four donors. (**E**) Nuclear localization of RELA following APRIL stimulation. Nuclear fractions were separated from unstimulated PBs at day 7 (*t* = 0) or stimulated with APRIL for 15, 30, 60, 120, 240, and 360 min; immunoblots for p65/RELA (upper) or histone H3 (lower) are shown. Data are representative of four donors. (**F**–**H**) Activation of AKT/FOXO1 pathway after APRIL stimulation. Day 7 PBs were unstimulated (*t* = 0) or stimulated with APRIL for 5, 15, 30, 60, 120, 240, and 360 min. Samples were probed for (F) AKT p-serine 473 (p-AKT S473) and total AKT; (G) AKT p-threonine 308 (p-AKT T308) and total AKT; and (H) FOXO1 p-threonine 24 (p-FOXO1 T24) and total FOXO1 as indicated. Data are representative of four donors (F–H). (**I**) Cytoplasmic and nuclear extracts (C and N above the lanes) from unstimulated (*t* = 0) PBs at day 7 or treated with APRIL for 120, 240, or 360 min were blotted for FOXO1 (upper), FOXO3 (middle), and tubulin (lower). Data are representative of four donors.

Having established conditions under which APRIL supported survival of both in vitro–generated and ex vivo–derived PBs and allowed maturation of these populations to the PC state, we were in a position to evaluate the downstream signaling pathways regulated during this response. We focused on the initial transition when the PB encounters the APRIL signal and evaluated downstream signaling pathway activation. APRIL stimulation activated MAPKs (Fig. 3A–C) with p38 and JNK activation sustained for 120 min following APRIL stimulation (Fig. 3B, 3C). In parallel, APRIL signals led to rapid IκBα phosphorylation and subsequent loss of IκBα protein (Fig. 3D) and nuclear translocation of RELA (Fig. 3E). AKT phosphorylation was also induced by APRIL stimulation (Fig. 3F, 3G). Although AKT S473 phosphorylation was more intense than that of T308, full activation of the pathway was supported by induced phosphorylation of FOXO1/3 with a kinetics consistent with the pattern of AKT activation (Fig. 3H). Both FOXO1 and FOXO3 were expressed in PBs. Whereas FOXO3 was primarily cytoplasmic, FOXO1 showed evidence of nuclear localization prior to stimulation with evidence of nuclear exclusion at later time points after APRIL treatment (Fig. 3I). Therefore, APRIL can drive a broad range of signaling responses at the PB transition point.

### APRIL drives a cell growth–related expression program

**FIGURE 4.**
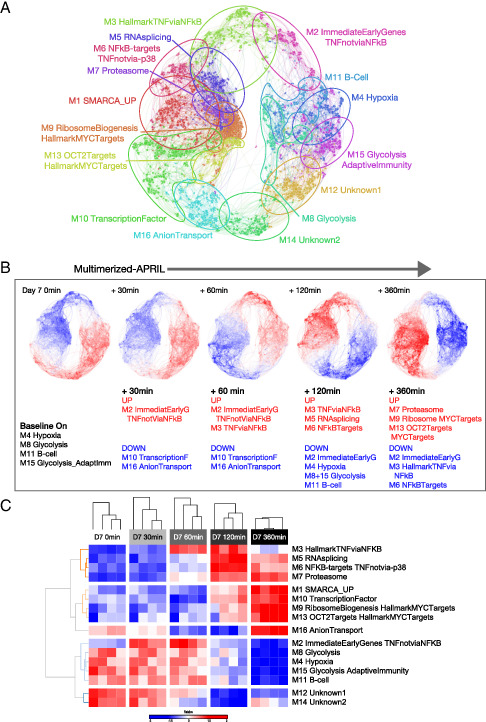
The transcriptional response of PBs to APRIL stimulation. (**A**) PGCNA network representation of the modular pattern of gene expression induced following APRIL stimulation of day 7 PBs during a 360-min time course. Network modules M1–M16 are color coded and are designated with a summary term derived from Gene Ontology and signature separation between network modules. For interactive version and lists of module genes and ontology enrichments, go to https://mcare.link/STC-APRIL. (**B**) Overlay of gene expression *Z* scores (blue [low] to red [high] color scale) for all genes in the network across the time course indicated by the arrow above the panel from left to right. Left panel shows unstimulated *t* = 0 followed by expression patterns at *t* = 30, 60, 120, and 360 min from left to right as indicated in the figure. Beneath each color-coded network selected upregulated and downregulated modules at each time point are indicated using module number and summary term (red indicates upregulated module expression, blue indicates downregulated module expression). (**C**) Summary representation of patterns of expression across all network modules as a heatmap showing median module expression value *Z* scores with a color scale (blue [−3 low] and red [+3 high]). Samples and modules are separated by hierarchical clustering. Module numbers and designations are shown on the right.

We next evaluated the overall impact of APRIL on gene expression in differentiating PBs. We applied a combination of gene expression time course, evaluating samples with RNA sequencing at 0, 30, 60, 120, and 360 min after APRIL stimulation, and PGCNA (24). We analyzed the data for differentially expressed genes across the time series using a likelihood ratio test (FDR < 0.01; for extended data, see online resources at https://mcare.link/STC-APRIL). The resulting 4615 genes were used to generate a gene correlation network that resolved into 16 modules (Fig. 4A and online resources). We analyzed the biology associated with these gene expression modules using gene signature and ontology enrichment analysis (Supplemental Fig. 2 and online resources), selecting a suitable summary term for each module from the observed enriched ontologies and signatures.

**FIGURE 5.**
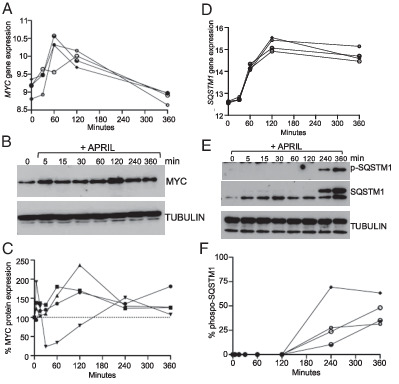
APRIL induces MYC and SQSTM1 expression. (**A**) Expression of *MYC* mRNA induced following APRIL stimulation with VST expression values derived from RNA sequencing expression profiling as in (Fig. 4 with individual donors shown with symbols at the indicated time points in minutes (*x*-axis). (**B**) Representative Western blot of *n* = 4 for MYC expression in day 7 PBs after APRIL stimulation, unstimulated (*t* = 0) or stimulated with APRIL for 5, 15, 30, 60, 120, 240, and 360 min as indicated. Upper panel, MYC; lower panel, tubulin loading control. (**C**) MYC protein expression quantified against tubulin loading control across an APRIL response time course as shown in (B) for four individual donors identified with unique symbols. Expression is normalized to 100% for all samples based on expression for each donor at *t* = 0. (**D**) Expression of *SQSTM1* induced following APRIL stimulation with VST expression values derived from RNA sequencing expression profiling as in (Fig. 4, with individual donors shown with symbols. (**E**) Representative Western blot of *n* = 4 for SQSTM1 expression after APRIL stimulation, unstimulated (*t* = 0) or stimulated with APRIL for 5, 15, 30, 60, 120, 240, and 360 min shown above the lanes. Upper panel, p-SQSTM1; middle panel, total SQSTM1; lower panel, tubulin loading control. (**F**) Quantification of p-SQSTM1 intensity as a proportion of total SQSTM1 normalized to tubulin.

To assess the kinetics of gene expression change, relative gene expression was overlaid on the network and assessed as a module expression value heatmap (Fig. 4B, 4C and online resources). This illustrated a wave of gene expression propagating through the network. The initial activation was observed in module M2, enriched for immediate early genes, and genes linked to TNF response signatures (Supplemental Fig. 3). This was followed by a further module linked to NF-κB signaling at 60 min (M3), which was sustained to 120 min, at which time it was joined by a wider diversity of NF-κB target genes (M6) and a module of genes enriched for factors involved in RNA splicing. Finally, by 360 min, as the initial signaling modules (M2, M3, and M6) waned, the secondary response was enriched for modules related to MYC (M9 and M13) and OCT2 targets (M13) along with the ribosome (M9) and proteasome (M7). Indeed, PBs both retained detectable MYC protein expression and showed evidence of induction following APRIL stimulation by 60–120 min (Fig. 5A–C). Among additional multifunctional genes induced by APRIL is *SQSTM1*, which plays roles in signaling, autophagy, and metabolic regulation. SQSTM1 was notably both induced by APRIL stimulation and became phosphorylated providing a potential site for upstream signal integration (Fig. 5D–F). Thus, the response to APRIL in primary human PBs propagates from immediate early genes and NF-κB response modules to drive gene expression related to cell growth and metabolism.

### APRIL and CD40L share similar effects on growth-related gene expression

The differentiation of PBs to PCs can be supported by a variety of conditions in vitro, potentially reflecting different types of niche conditions that a PB may encounter in vivo. Differences in niche condition may impose distinct patterns of gene expression on the differentiating PC (3, 6). To directly address how APRIL-mediated gene expression differs from that induced by other potential niche signals, we compared the gene expression responses of PBs to stimulation with IFN-α, SDF1, APRIL, or sCD40L at 1 and 6 h.

**FIGURE 6.**
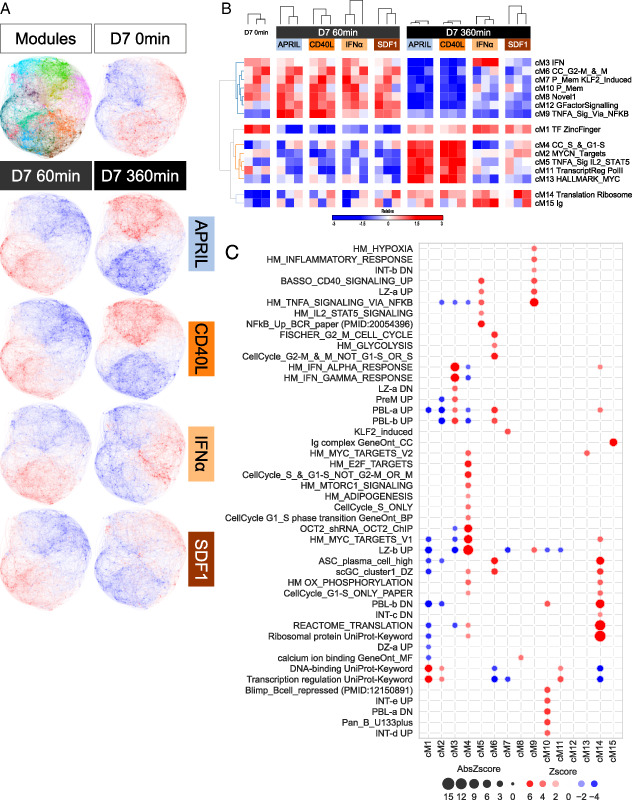
APRIL and CD40L share induction of NF-κB responses and growth-related programs. (**A**) Network analysis of gene expression changes following stimulation of PB with APRIL, CD40L, IFN-α, or SDF1. Network visualization shows module structure (left) or overlay of baseline gene expression at *t* = 0 (blue [low] to red [high] expression *Z* score color scale) (right) beneath this expression changes in response to the indicated stimuli are shown overlaid onto the network with the order APRIL, CD40L, IFN-α, and SDF1 (top to bottom) at 1 h (left panels) or 6 h (right panels). (**B**) Summary representation of patterns of module expression encompassing all modules of the network as a heatmap. Shown are median module expression values of each module as median *Z* scores with a color scale (blue [−3 low] and red [+3 high]). Samples are separated by time point and stimulation condition as indicated above the heatmap, and modules are hierarchically clustered and labeled on the right. (**C**) Bubble plot of enrichment of representative Gene Ontology and gene signature terms between modules of the network. Enrichments are shown on a blue (depletion) to red (enrichment) scale as indicated below the figure; size of bubble reflects absolute *Z* score of enrichment or depletion.

Addressing the overall differential gene expression between these stimuli using network analysis, a total of 15 gene modules were identified in this comparative analysis (Fig. 6A and online resources at https://mcare.link/STC-APRIL); modules are designated cM1–cM15 to disambiguated from modules described for APRIL response alone. Clustering of samples according to module expression values separated both on time point of stimulation and stimulus type (Fig. 6B). The individual samples differed at 0 h with respect to baseline expression state, consistent with subtle differences in differentiation between donors, in particular for modules relating to the G_2_/M phase of the cell cycle (cM6) and genes related to plasma membrane components and KLF2 targets (cM7) (Fig. 6B, 6C). At 1 h a coordinated induction of early response gene modules was observed separating into two patterns (cM12 and cM9). Module cM12 was induced to a broadly similar extent by each stimulus with IFN-α being the weakest, and with modest variation between donors. This module included a number of immediate early and growth factor response genes and overlapped with modules previously identified for SDF1 responses in PBs (genes such as *CD69* and *NR4A2*) and growth factor responses in cancer (genes such as *EGR1/3, FOS*, *FOSB*, and *IER2)* (Supplemental Fig. 4) (6, 24). Module cM9 was induced strongly by sCD40L and APRIL with modest induction by SDF1 and weakest response for IFN-α and was enriched for hallmark signatures of hypoxia, inflammatory response, and TNF signaling via NF-κB (genes such as *ATF3, BCL2A1*, *CD83*, *DUSP2/4/5*, and *ICAM1*). At 6 h, differences between the stimuli became more profound. Coordinated loss of expression in modules present at early time points was observed (cM6, cM7, cM8, cM10, cM12). Despite robust early responses, SDF1 generated the least distinctive pattern of expression at 6 h, whereas IFN selectively induced two modules, that is, cM3 and cM15. Of these cM3 included most IFN-responsive genes, while additionally being enriched for genes associated with the PC state such as *CD38*, *IRF4*, and *XBP1*. However, at 6 h the most distinct pattern was the response to sCD40L and APRIL. These stimuli induced several gene modules (cM2, cM4, cM5, cM11, and cM13) that were enriched for the following: MYC target genes (cM2, cM4 and cM13); OCT2, E2F, and MTORC1 targets and genes expressed in light zone germinal center B cells (cM4); hallmark signatures of NF-κB target genes (cM5); and regulation of transcription (cM11). cM4 genes also overlapped with cell cycle signatures including those related to the G_1_/S transition including *CCND2* and *CCNE2*.

We conclude that the initial response induced by APRIL niche signals is largely shared with the response to CD40L, which in both cases propagates from immediate early and NF-κB target genes into modules characteristic of MYC-, MTORC1-, and E2F-associated gene expression linked to cell growth and G_1_/S phase transition. This pattern of response is not observed following acute SDF1 and IFN stimulation and was also not observed in our previous studies of extended responses to SDF1 or IFN at later time points (3, 6). Thus, TNFSF/TNFRSF related niche signals induce a distinctive pattern of cellular activation accompanying the support for cell survival.

### APRIL avoids activating B cell programs

**FIGURE 7.**
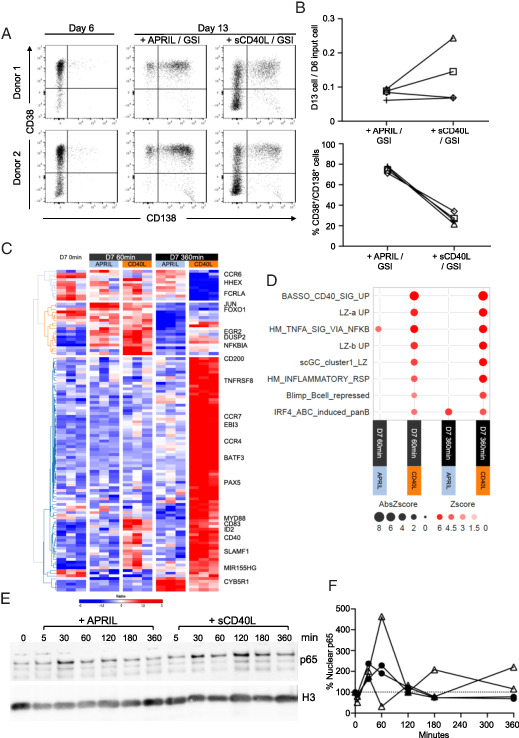
APRIL and CD40L differ in terms of population phenotype and regulation of activated B cell–like program. (**A**) Representative phenotypes for cells from two donors at day 6 (left panels) or day 13 of culture with additional of APRIL/GSI (middle panels) or sCD40L/GSI (right panels) along with supportive cytokines IL-6 and IL-21. Shown are scatterplots for expression of CD38 (*y*-axis) against CD138 (*x*-axis). (**B**) Upper panel shows recovered cell number at day 13 for PBs cultured from day 6 with APRIL/GSI (left) or sCD40L/GSI (right) (*x*-axis); *y*-axis displays day 13 (D13) cells as fraction of day 6 (D6) input. Lower panel shows percentage of CD38^+^/CD138^+^ cells at day 13 as a percentage of viable cells (*y*-axis) for PBs cultured from day 6 with APRIL/GSI (left) or soluble CD40L/GSI (right) (*x*-axis) along with supportive cytokines IL-6 and IL-21. Four individual donors are identified by symbols. (**C**) Genes significantly differentially expressed between APRIL and CD40L at t = 1 h and *t* = 6 h are shown as a heatmap with *Z* score of gene expression (−3 blue to +3 red) with expression at day 7 *t* = 0 baseline conditions on the left, and then day 7 + 1 h (D7 60 min) and 6 h (D7 360 min) for the conditions ordered for each time point APRIL (left columns) and CD40L (right columns). (**D**) Bubble plot of selected gene signatures significantly enriched among genes differentially regulated by CD40L or APRIL at *t* = 1 or 6 h. Enrichments are shown on a *Z* score scale as indicated below the figure; size of bubble reflects absolute *Z* score. (**E**) Western blot of nuclear extracts for a time course of PB stimulation with APRIL (left) or sCD40L (right) at *t* = 0, 5, 15, 30, 60, 120, 180, and 360 min blotted with p65/RELA (upper) and histone H3 loading control (lower). Data are representative of two independent experiments. (**F**) Quantitations of nuclear p65/RELA localization shown in (E) normalized to histone H3 (nuclear loading) response for individual donors are shown with symbols for APRIL (●) and CD40L (▵).

Both APRIL and sCD40L signals drive a gene expression pattern consistent with cellular growth, but these stimuli ultimately generate significant differences in the phenotype of cells surviving on subsequent culture. Thus, APRIL selectively supported PCs while CD40L enhanced survival of a mixed population of B cells and PCs (Fig. 7A, 7B). We therefore asked whether there were differences in gene expression that might explain the difference in response. Although the number of genes differentially regulated was small at 1 h, that is, 14 genes upregulated for sCD40L and 2 genes upregulated for APRIL, this became more substantial by 6 h, that is, 92 genes upregulated for sCD40L and 17 genes upregulated for APRIL (Fig. 7C). Indeed, the 17 genes relatively upregulated in APRIL conditions at 6 h primarily reflected more profound repression in response to sCD40L relative to baseline, rather than induced expression in response to APRIL, and included *FOXO1* and *ID3*. Genes upregulated by sCD40L primarily represented induced expression relative to baseline and included genes linked to CD40 and NF-κB responses in B cells overlapping with signatures of light zone germinal center B cells (Fig. 7D). Indeed, CD40L provided a more potent stimulus of the NF-κB pathway than APRIL as assessed by nuclear RELA localization under the conditions tested (Fig. 7E, 7F). However, differences in gene expression included several key regulators of the B cell and activated B cell state such as *PAX5*, *BATF3*, *CD83*, *miR155HG*, *SLAMF1*, *TNFRSF8* (CD30), and *CD40* itself (Fig. 7C, Supplemental Fig. 4). This argues for a qualitative as well as quantitative difference to the CD40L response relative to APRIL, with CD40L driving gene expression linked to maintenance of the B cell state within the PB population, whereas APRIL does not. We conclude that at the PB transition APRIL supports PC survival while avoiding support for the B cell state.

## Discussion

The generation and maintenance of long-lived PCs from transitional PB populations is dependent on localization to microanatomical niches that provide suitable signals for survival (4). Among niche signals that have been defined as important for long-lived PC generation in vivo are signals delivered by the TNFSF member APRIL (12). In this study, we have used an in vitro model system to address the signaling and gene regulatory response in human PBs induced by encounter with APRIL. Our data demonstrate that the acute signaling responses are diverse, including activation of canonical NF-κB, p38, and JNK MAPK as well as the AKT/FOXO pathway. This pattern recapitulates the pattern demonstrated for BCMA activation in cell line models (7, 18). This confirms that broadly diverse pathways are activated by this factor in differentiating primary human ASCs.

The precise pathways responsible for signal propagation at the plasma membrane following exposure to APRIL remain to be determined. We confirmed the observation of Laurent et al. (10) that γ-secretase inhibition maximizes surface BCMA expression. This increase in BCMA expression translated into enhanced APRIL-induced survival responses. In conjunction with the existing literature it is therefore most likely that the dominant receptor for the APRIL response in this model is indeed BCMA. Although TACI expression is not substantially impacted by γ-secretase treatment, it is expressed in PBs, and thus a contribution to signal propagation is likely in the model. Our analysis shows that the APRIL signal propagates into successive waves of gene regulation. These follow a canonical pattern of immediate early, delayed early, and secondary response gene regulation (27). The most immediate responses are the control of coordinated modules of genes shared with the TNF response that have been attributed to NF-κB–independent signaling, and most likely relate to MAPK pathway induction (28). These are closely followed by modules of genes that together are enriched for targets of NF-κB, and that are consistent with the observed RELA activation. Although the peak of detectable nuclear p65/RELA translocation in response to APRIL was relatively transient, evidence of nuclear translocation remained detectable at 60 min. The observed impact on expression of NF-κB target genes was sustained to 120 min with subsequent decay at 360 min. This is consistent at the transcriptional level with a substantial contribution of this pathway to the overall response but is likely also to reflect synergy between NF-κB and other transcription factors. The AP-1 family downstream of MAPK activation may provide one explanation that can cooperate with NF-κB for DNA binding (29). Indeed, recently it has been suggested that relatively transient RELA activation may prime loci for subsequent more prolonged expression driven via other pathways such as ERK-regulated AP-1 activation (30). Unexpectedly, given the differentiation stage, the response to APRIL propagates further into secondary response genes that relate to ribosome-, MYC-, and cell growth–related gene expression patterns. Our previous studies of alternate niche signals supporting PC differentiation had failed to identify such a pattern of gene expression (3, 5, 6), suggesting that this was a specific feature of the response to APRIL or to a TNFSF/TNFRSF survival signal. This led to the direct comparison of APRIL to alternate potential niche signals IFN-α, SDF1, and sCD40L. Although each niche signal was capable of activating immediate early genes, only APRIL and sCD40L drove a substantial component of gene expression that culminated in MYC-, E2F-, and MTORC1-related gene expression, including features related to cell growth and G_1_/S transition. Thus, the TNFSF-mediated response in PBs differed substantially from the survival signal delivered by IFN-α or the niche homing signal SDF1. The TNFSF members, however, also differed with sCD40L additionally driving gene expression related to the activated B cell state. We consider at least two possibilities for this difference that might either be attributable to heterogeneity in the PB culture and variances in the differentiation state of cells able to respond to these two signals, or due to differences in the way the resulting signals are interpreted within cells of similar differentiation states. Single cell–based approaches may provide a means to resolve such interesting questions of heterogeneity in response to niche signals in the future. Considering the focus in the present study on the shared response induced by both TNFSF members we note that at a functional level this includes expression features that would be consistent with the recently proposed concept of cellular refueling observed in light zone germinal center B cells at the point of follicular helper T cell encounter (31). We conclude that support delivered by APRIL for PC survival is delivered by a broad range of signaling pathways and converges on growth-related gene expression while being uncoupled from support for the B cell state. This suggests that the recently proposed cellular refueling model could be extended to explain APRIL-mediated PC survival.

## Supplementary Material

Data Supplement
