# Supplementary material for: APRIL Drives a Coordinated but Diverse Response as a Foundation for Plasma Cell Longevity
Source: J Immunol. 2022 Sep 1;209(5):926–37. doi: 10.4049/jimmunol.2100623 (PMC7613700; doi:10.4049/jimmunol.2100623)
Supplement: Data Supplement [file JI_2100623.zip › ji_2100623_supplemental_1.pdf]

Supplemental Figure 1 (accompanies Figure 2)

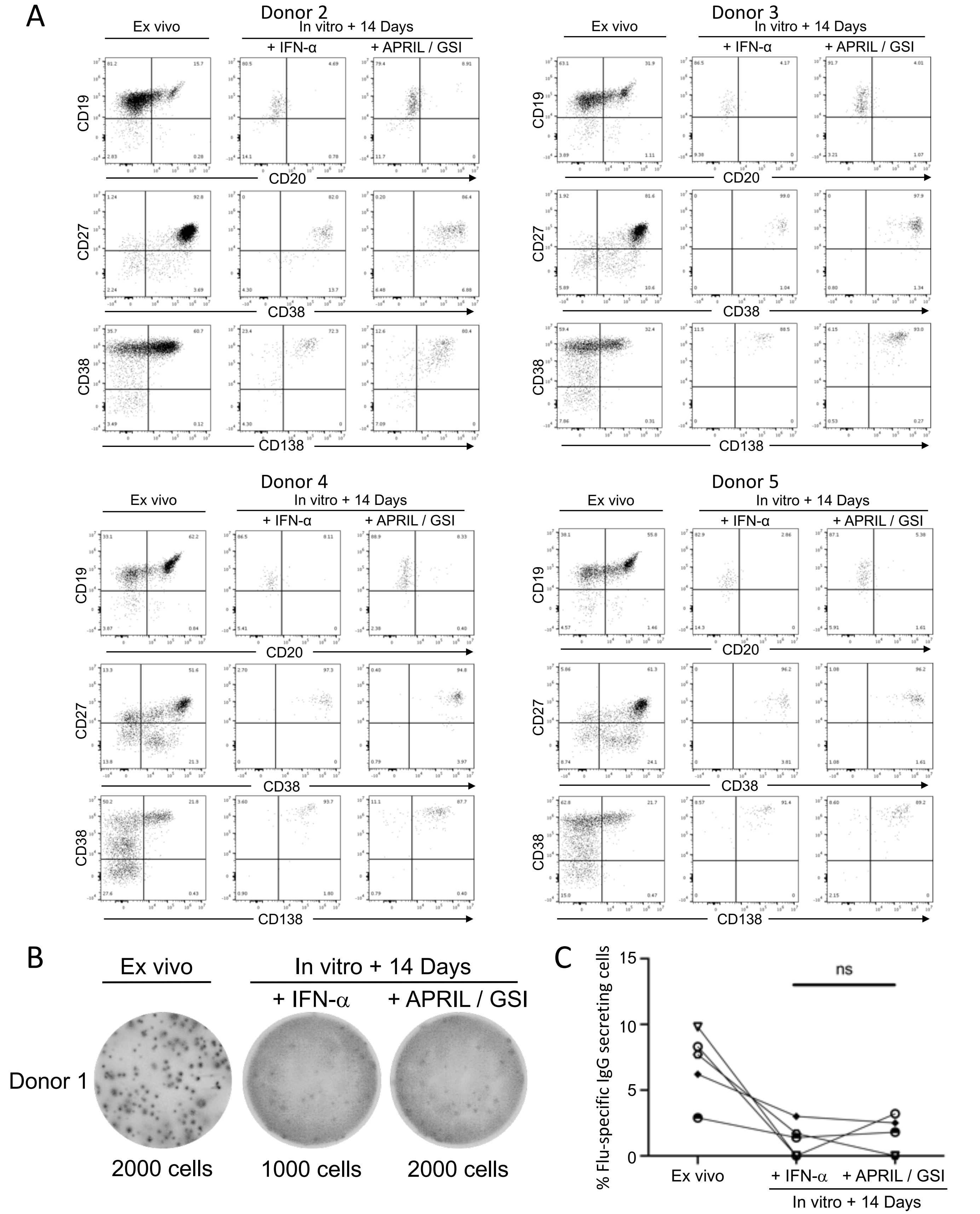

Supplemental Figure 1 (accompanies Figure 2). APRIL supports ex vivo PB maturation. (A) Phenotypes of cells isolated ex vivo at day 7 after influenza vaccination left panels (ex vivo), or after 14 days of in vitro culture with IL6 and either IFNα (middle) or APRIL/GSI (right) panels. Plots from top to bottom show CD19/CD20, CD27/CD38 and CD38/CD138 as indicated for 4 individual donors (donors 2-5). **(B and C)** Representative ELISpots for influenza specific ASCs for cells isolated at day 7 post vaccine response (ex vivo), or after 14 days of in vitro culture with IL6 and either IFNα (middle) or APRIL/GSI (right) panels, equivalent to day 21 post influenza vaccination (B) and quantitation (C). Numbers of cell seeded per well are shown below. Cells were incubated on plates for 16-20 hours in IMDM containing either standard amounts of IL6 and IL21 (Control, D7) or IL6 with either IFNα or APRIL/GSI (D21).

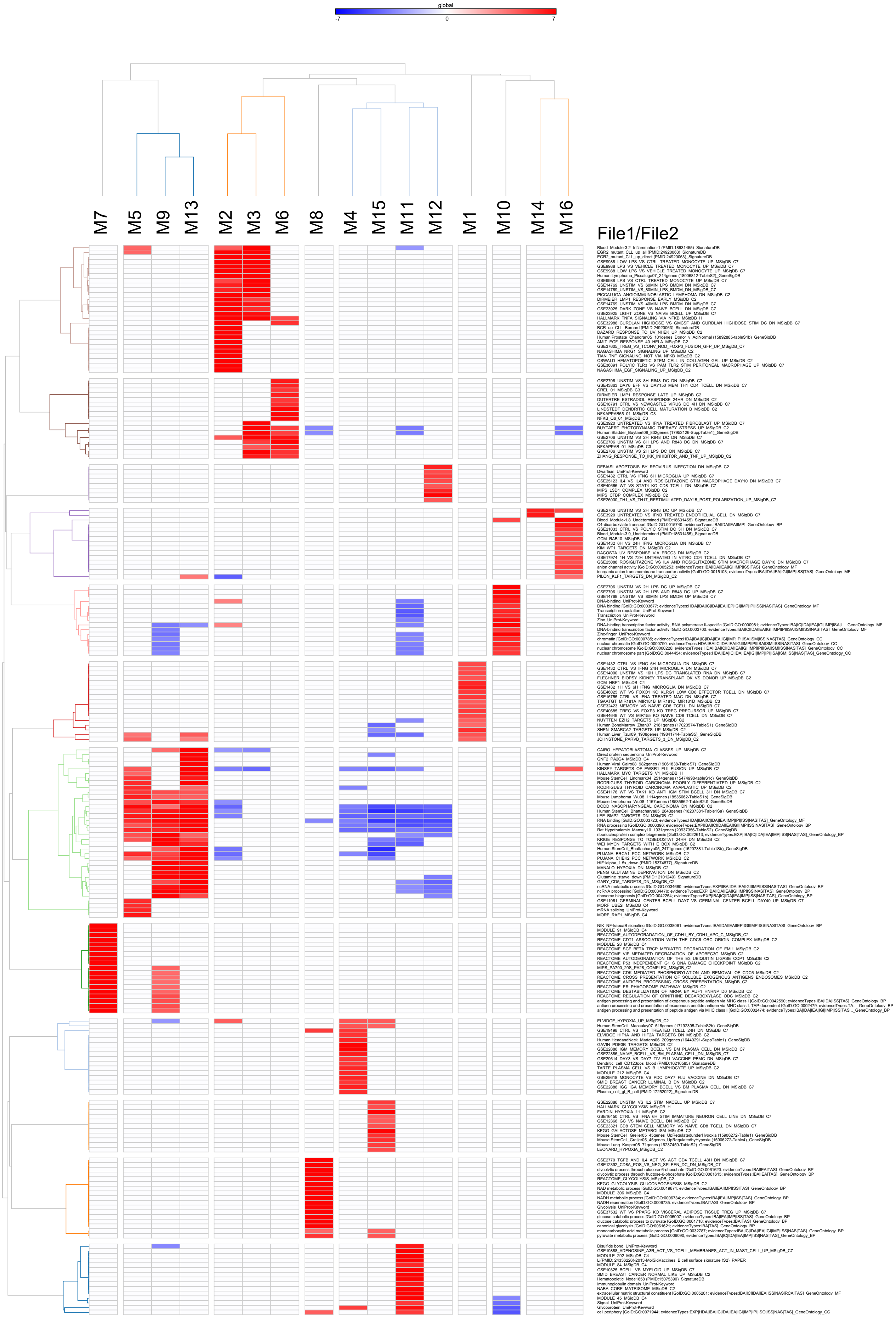

Supplemental Figure 2 (accompanies Figure 4). Gene ontology and signature enrichments for gene modules of the PB response to APRIL. Heatmap of gene ontology and signature term enrichments linked to the PGCNA modules of the time course network analysis for APRIL response (signatures were pre-filtered to p-value <0.001 and ≥ 5 and ≤ 1000 genes; selecting the top 15 most enriched signatures per module). For full signature enrichment lists, please see Supplemental Table 3. Modules are shown along the x-axis, and signature terms along the y-axis. Signature terms and modules are hierarchically clustered to illustrate relationships. Enrichment (red) and depletion (blue) of signatures are shown on colour scale of z-score.

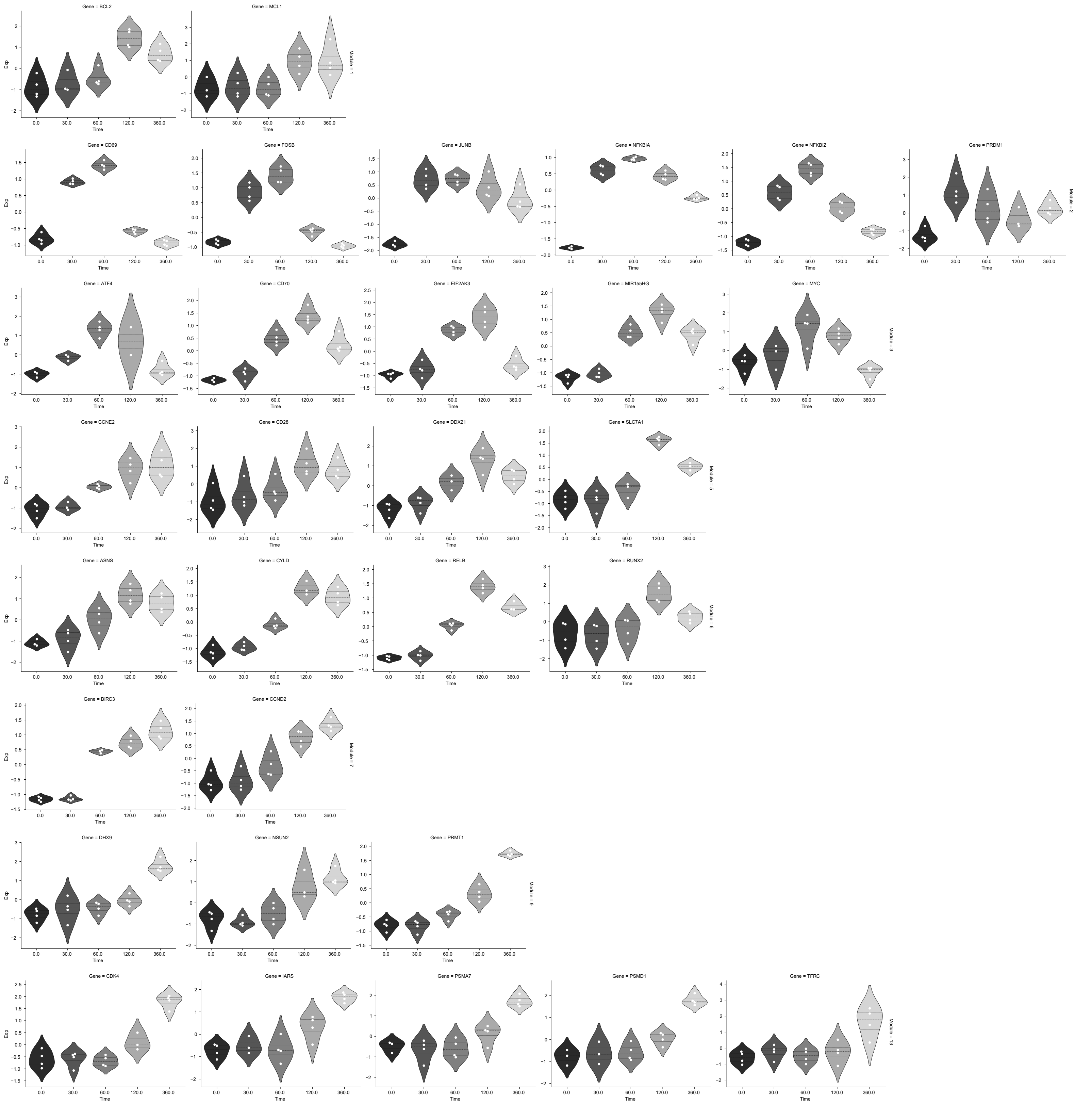

Supplemental Figure 3 (accompanies Figure 4). Selected gene expression responses following APRIL stimulation. Shown are violin swarm plots for patterns of gene expression observed for selected genes across a range of modules following APRIL stimulation. The range of expression is shown as an expression z-score, with interquartile ranges and individual data points. Genes and module memberships are indicated above/right of each panel.

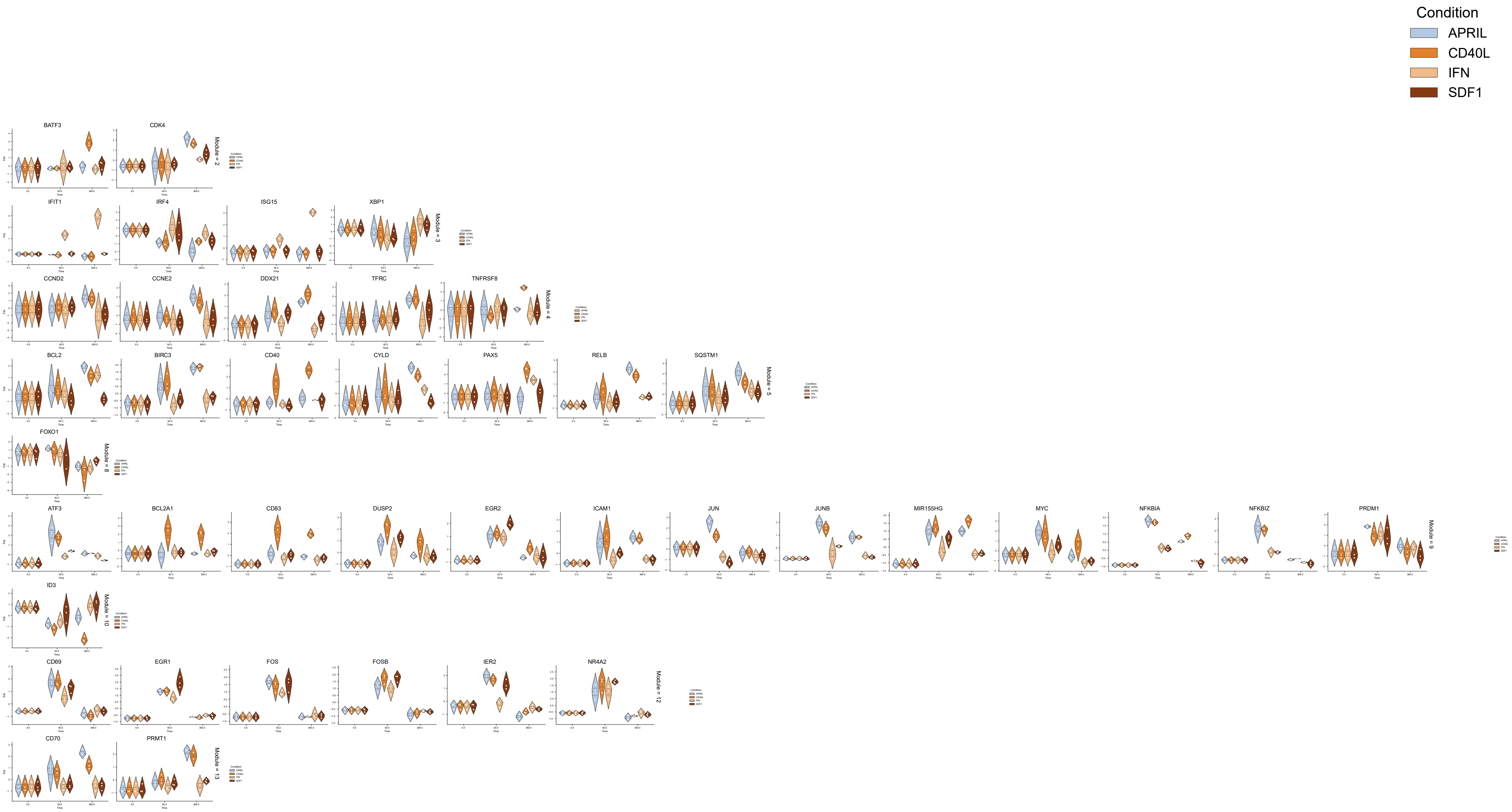

Supplemental Figure 4 (accompanies Figure 6). Selected gene expression responses following APRIL, CD40L, IFNα or SDF1 stimulation. Shown are violin swarm plots of patterns of gene expression observed for selected genes across a range of modules following stimulation with different niche conditions. The range of expression is shown as an expression z-score, with interquartile ranges and individual data points. Genes and module memberships are indicated above/right of each panel.
